# What factors affect paramedics’ involvement of people with dementia in decisions about their care? A qualitative study

**DOI:** 10.29045/14784726.2021.3.5.4.1

**Published:** 2021-03-01

**Authors:** Esme Choonara, Julia Williams

**Affiliations:** London Ambulance Service; University of Hertfordshire; University of Hertfordshire

**Keywords:** decision making, dementia, paramedics

## Abstract

**Background:**

Paramedics are frequently called to people with dementia, but decision making can be challenging due to lack of information or difficulties in assessment. Best-practice dementia care should be holistic and involve people with dementia in decisions as far as possible.

**Aims:**

To explore how paramedics make decisions when attending people with dementia, with a particular focus on factors that impact on how, and to what degree, paramedics involve people with dementia in these decisions.

**Methods:**

A generic qualitative research approach was used. Data were collected through semi-structured individual interviews with seven paramedics. The interviews were recorded and transcribed verbatim and subsequently analysed using thematic analysis.

**Results:**

Four themes were identified that all touched on challenges to delivering person-centred care. Themes identified were: 1) paramedics’ differing approaches to assessing capacity and making best interest decisions; 2) communication and developing a rapport; 3) interconnections with others important to the person with dementia; and 4) the impact of paramedics’ values and attitudes.

**Conclusion:**

The involvement of people with dementia is sometimes limited by medical, social or clinician-dependent factors. This study highlights how paramedics’ values and communication skills influence their interactions with people with dementia. As the paramedic role evolves, there is an opportunity to embed person-centred care in practice and to ensure that education equips paramedics with the skills and ethical frameworks needed to deliver high quality dementia care.

## Introduction

There are more than 50 million people living with dementia worldwide (Alzheimer’s Disease International, 2019), including around 850,000 people in the UK (Prince et al., 2016). Emergency ambulances are frequently called to older people with dementia due to high rates of complex needs and co-morbidities (Buswell et al., 2016; Schubert et al., 2006). However, decision making in this context can be challenging for paramedics due to lack of information, limited assessment tools and communication difficulties (Simpson et al., 2017; Voss et al., 2017).

High quality dementia care requires a holistic person-centred approach that accepts that every person with dementia is unique (Brooke & Stiell, 2018). This approach is based on the idea of ‘personhood‘ pioneered by Kitwood (1997), and emphasises the reinforcing of a sense of self for people with dementia, while delivering care that enriches their social world. This concept of personhood has underpinned the emphasis on shared decision making in policies and guidelines in dementia care for over a decade (Mitchell & Agnelli, 2015) and also informs the central principles of the Mental Capacity Act 2005 (Hughes & Baldwin, 2006).

Best-practice guidelines state that healthcare professionals should involve people with dementia in decisions affecting their care as far as possible (Department of Health, 2009; NICE, 2016). However, many people with dementia still experience care that is poor quality, distressing and/or undignified (Grosvenor et al., 2017).

Although there is a growing body of work looking at paramedics’ decision making, there is little specifically exploring this in relation to people with dementia (Buswell et al., 2015; Voss et al., 2020). A significant mixed-method study, the Homeward Project (Voss et al., 2017), has investigated how older people with dementia use ambulance services and how paramedics make decisions about whether to take these people to hospital. The qualitative part of this study has highlighted that while paramedics often prefer not to convey people with dementia to hospital, difficulties in assessment and history-taking can make these decisions challenging (Voss et al., 2020).

This study aimed to explore how paramedics make decisions when called to people with dementia, but with a particular focus on understanding paramedics’ interactions with people with dementia and examining factors that influence paramedics’ involvement of people with dementia in decisions about their care.

## Methods

### Study design

The study was conducted using a generic qualitative research methodology, an approach that offers the flexibility and pragmatism needed to address clinical problems in emergency medicine (Cooper & Endacott, 2007), facilitating the utilisation of various qualitative methods without being aligned to one specific underpinning theoretical philosophy (Williams, 2012). The study was informed by the researcher’s commitment to the concept of ‘personhood’ in dementia care (Kitwood, 1997).

This study did not require Health Research Authority (HRA) approval as it met their exemption criteria, including: being part of an educational programme (MSc); taking place in England at a single NHS site; and not needing review by an NHS ethics committee. Therefore, local ethics review and research governance permissions were obtained. Ethical approval for the study was granted by the University of Hertfordshire Health, Science and Technology Ethics Committee with Delegated Authority (HSK/PGT/UH/03761), and permission to recruit participants was given by the London Ambulance Service (LAS) Clinical Audit and Research Unit.

### Recruitment and sample

Seven participants were recruited via an internal LAS online staff newsletter. This sample size balanced the need for collection of in-depth data with the practical limitations of a small project. Inclusion criteria were Health and Care Professions Council (HCPC) registered paramedics with at least one year’s experience post registration. Exclusion criterion was anyone already known to the researcher.

Participants included five females and two males with between one and 25 years of front line emergency ambulance experience (Table 1). Participants received an information sheet and gave written consent prior to data collection.

**Table 1.** Characteristics of participants.

Paramedic	Gender	Role	Front line ambulance experience (years)	Paramedic experience (years)
1	Female	Newly Qualified Paramedic (NQP)2	1.5	1.5
2	Female	NQP2	1.5	1.5
3	Female	NQP2	1.5	1.5
4	Female	NQP2	1.5	1.5
5	Female	Paramedic	10	6
6	Male	Paramedic	>20	>20
7	Male	Paramedic	>15	3

### Data collection

Semi-structured interviews were conducted and recorded by the researcher, lasting between 40 and 80 minutes. These mostly took place at the participants’ ambulance station or another LAS premises, with one interview taking place at the participant’s home.

Audio recordings were transcribed verbatim by the researcher. Field notes were taken immediately after interviews and during transcription to note inflexions and non-verbal communication and to reflect on emerging concepts.

### Analysis

The researcher analysed the data using thematic analysis informed by the approaches of Braun and Clarke (2006) and Merriam and Tisdell (2016). Each transcript was coded using QSR International’s NVivo 12 qualitative data analysis software (NVivo, 2018) to organise the data. Data were continuously compared as new codes were identified and refined (Percy et al., 2015). A reflexive diary was maintained to reflect on meanings in the data and changes in coding, and to scrutinise the researcher’s interpretation. Codes were grouped thematically with reference back to the research questions (Merriam & Tisdell, 2016), leading to the identification of four main themes.

A summary of the findings was sent to the participants for member checking in order to ensure the researcher had not misrepresented the data and to increase the credibility of the research (Maxwell, 2013). Three participants replied, confirming the themes and stressing areas they thought were of particular importance. This was integrated into the final report.

## Results

Four interrelated themes were identified from the data, all of which touch on the challenges of delivering person-centred care.

### Theme one: differing approaches to capacity and best interest decisions

Five participants described a structured approach to assessing the capacity of a person with dementia. However, methods varied, with some using a form with integrated prompts provided by their employer and others being more focused on informal conversation. One participant stated that she used a mini-mental test to assess capacity.

While establishing whether someone had capacity was seen as important by six of the participants, one did not mention it at all until prompted, and said that it was not central to her approach as decisions could be made collaboratively.

Usually you can come to an agreement without having to even think about the capacity tool or the Capacity Act or anything. (Paramedic 5)

All participants felt that where people had capacity to make their own decisions, the process was quite straightforward. However, where patients were assessed as lacking capacity, this involved a complex process of ‘best interest‘ decision making.

These decisions were reached by weighing clinical factors, social and safety concerns and any advanced care planning against the views of family, carers and the person with dementia. The availability of community teams and GPs who were willing to take responsibility for ongoing care was also mentioned by a majority of participants as a factor affecting their decisions.

There was a variance in the weight given to the wishes of the person with dementia where they were found not to have capacity, with four participants concentrating on making the best interest decisions *for* the patient and three trying to involve the patient.

In my mind shared decision making should be for everyone. It shouldn’t just be for people with capacity because at the end of the day, even if you don’t have capacity they may have had strong feelings about certain aspects of their care. (Paramedic 1)

Best interest decisions for all participants also involved an acknowledgement that hospital carries risks for older people, especially those with dementia.

I mean with any elderly person, anyway, I don’t think A&E is particularly helpful in a lot of cases, but more so with someone with dementia/Alzheimer’s. It’s just it’s a break away from their routine and it’s just something that can set them back so much. (Paramedic 5)

However, two participants, despite reluctance to convey, felt that hospital was often in the best interests of the patient for clinical reasons.

You know, if there’s a doctor or a geriatrician there that is better able to communicate with someone with dementia and more experienced, then they’re going to get better care. So I think often times it is in their best interest for us to take them to hospital when we can’t establish what’s actually wrong. (Paramedic 3)

### Theme two: communication and building a rapport

Participants all talked about building trust and a rapport with patients. Although participants discussed difficulties that can arise from cognitive impairment in people with dementia, they also recognised that communication was affected by clinicians’ skills and approaches.

Some people just have a knack for it – a way of trying to communicate. (Paramedic 6)

Participants unanimously tried to adjust their communication strategies to create greater trust and reduce distress, often by spending time engaging patients in broader conversations about their lives and their environment.

Maybe it’s a question of putting people at ease … you know, you talk to them ask them about their family and their children and they’ll happily talk away and then all of a sudden you find they’re talking to you about all sorts. (Paramedic 6)

However, one participant also stressed that she would spend less time, not more, trying to engage with a person with dementia if that person was unwell and lacked capacity.

In someone with dementia, then you know it’s harder to have that conversation and you know, at the end of the day you’re wasting time and they’re becoming more unwell, so often I just, you know, make them go to A&E. (Paramedic 3)

A majority of the participants described good communication and rapport building as intrinsic to treating a person with dignity and respect.

We have to develop a rapport and a trust so they need to feel that you’re working in their best interests. So that’s talking to them. Are you listening to what they want? (Paramedic 7)

Especially dementia patients, involving them I think is really important – for rapport as well, which I keep coming back to, but it is such a massive thing … They still have feelings, they’re still human. (Paramedic 4)

However, trust was also seen by four participants as important in persuading a person with dementia to go along with the paramedic’s decision.

You need to gain that person’s trust first and if they’re agitated and distressed, you know often they’re frightened of you so they are not freely going to come to hospital with you. (Paramedic 3)

All participants attempted to enlist others to help with communication – in particular families and carers. Two participants also mentioned being alert to non-verbal communication, including behavioural changes or other ways of expressing wishes.

It’s more, it’s yeah, so it’s not always that they clearly express they don’t want to go to hospital. It’s the patients that just don’t want to move. They want to stay in their chair. (Paramedic 6)

Three participants compared the way they adapt their approaches to people with dementia to working with children.

You’re trying to adapt your approach to the patient like you would with a kid, sometimes. That sort of, maybe I infantilise sort of the more severe cases of dementia a bit more. (Paramedic 1)

They teach you when you go to children to kind of be at their level and to talk in certain ways and non-threatening ways and things like that. So you could bring that to the dementia patient and you know, it’s about how you communicate. (Paramedic 6)

### Theme three: interconnections with others

Participants all saw people with dementia as connected to a wider group of people, primarily involving family, but also paid carers, friends and neighbours and other healthcare professionals. Decision making and communication were unanimously described as not just involving the patient, but negotiating with this wider group of people.

Look at everybody’s needs – it’s not always a single patient. It’s often in the interconnectivity between all the parties involved. (Paramedic 7)

Well-being was seen as particularly tied to family relationships, and participants all described ways in which they sought the views and opinions of family members. However, five participants also reported challenges associated with relatives who may be distressed or have conflicting wishes to the person with dementia.

I think you have to be careful about why a family is wanting a particular thing to happen. (Paramedic 1)

Paid carers in a person’s own home or a care setting were seen by a majority of participants as a less reliable source of information or support than family, although an important part of the social context.

Quite often unfortunately the carers don’t really know the patient very well. Like they can read the notes, but they don’t just they don’t know them personally. (Paramedic 5)

With carers, you don’t know how long they’ve had a relationship with this person. They might have been on with them for a week and they’ve said all week they’ve been fine, but that whole week they haven’t been their normal selves so you can never really trust the carers too much. (Paramedic 2)

It was widely felt that social care provision is inadequate and that this limits what options are available to people with dementia.

It’s a chronic problem isn’t it? It’s like Social Services funding isn’t there enough, there isn’t enough carers, there isn’t enough funding going into that social support. (Paramedic 1)

Although all participants said they tried to enlist family members and carers to help reassure or communicate with people with dementia, four participants said that at times family members could be a barrier to involving the person with dementia in decisions.

I think the main difficulty is usually family members that are on scene as well. They’re either, I seem to encounter like two kinds of family members. One, that they don’t want the patient to know that they have dementia, so they don’t even mention it … Or you walk in and the first thing they say is, ‘They won’t understand anything. They’ve got dementia’. (Paramedic 5)

### Theme four: paramedics’ values in relation to people with dementia

Participants unanimously expressed a wish to act in ways that minimised distress for people with dementia and were concerned that strangers, hospital environments, ambulance journeys and medical procedures could provoke fear and disorientation.

You put them in a bed, you put the rails up, and then you go … I can’t imagine what that must be like, if you don’t know what’s going on … and then they’ve got a bunch of new people coming in and out, in and out, blood pressures every 10 minutes, bloods, sharp needles, other people in the department, it’s busy, it’s loud. (Paramedic 4)

Participants were all positive about attending people with dementia and felt no trepidation about this. However, five also expressed more negative emotions associated with dementia that included sadness, worry, helplessness and frustration.

Although there was little explicit discussion of values, all participants in some way saw treatment of the person with dementia as reflecting on their own character.

They always say you have to empower the patient … If anything, at the end of the day, it tells you something about yourself that you don’t consider their being empowered means anything. (Paramedic 2)

Six of the participants contrasted their own approaches to that of their colleagues.

I think I still have a bit more compassion because I’m newer, so I think people definitely get burnt out. Especially in the caring kind of aspect. So I guess there is a little bit of discrepancy when I’m with another crew mate between what we should do, what I would like to do and what they’re going to do. (Paramedic 2)

I think we’ve got lots of relatively inexperienced staff at the moment. So they don’t really see that process of the calm, relaxed approach … like this is just going to take two hours and we’re just going to have to sit down and have a cup of tea with his patient to build some trust in them. (Paramedic 7)

The involvement of people with dementia in discussions and decisions was seen as important to their dignity. Participants unanimously stressed that they treat each person as an individual. They described offering choices within parameters – so if a decision had been made that the person with dementia needed to go to hospital, they might be offered a choice of which hospital, or what to bring with them, or given some other control over the process.

It’s just about dignity, really, and respect for what they want to do. If they want to stop and chat to their neighbour for 10 minutes and explain where they’re going, I’m fine – do it, doesn’t bother me. (Paramedic 2)

A majority of participants said they felt that the protocol-led nature of paramedic practice was not always helpful when attending a person with dementia.

There’s like protocols … So everything you do, you kind of you can evidence that you’ve done it or there’s like a tick box exercise for it. Whereas with dementia you can’t do that because every single person is different and every type of dementia is different. So it’s quite hard to then use your like tick box training, and move it across to dementia patients. (Paramedic 5)

A majority of participants also expressed concerns that their training neglected the sorts of skills needed for these complex decisions. They tended to learn ‘on the job’ or from colleagues and there was some frustration that education was not felt to be keeping up with the changing role of paramedics.

## Discussion

Although this study was framed around decision making in general, most participants focused on dilemmas around whether to convey people with dementia to hospital. These were seen as the most complex decisions and the most likely to involve conflict between the wishes of the paramedic and the person with dementia and/or their family and carers. However, in contrast to previous research that found that paramedics view hospital as the safest option for both patient and paramedic (Ingram et al., 2019; Simpson et al., 2017), all of the paramedics in this study stated a preference not to convey people with dementia to hospital. This reinforces a similar recent finding by Voss et al. (2020).

This reluctance to convey was based on participants’ desire to reduce distress and disruption to the person with dementia, a belief that most older people do not want to go to hospital and an understanding of the potential adverse effects of hospital admissions for older people (Boltz et al., 2010). Despite the majority of participants describing scenarios where they felt conveying someone with dementia to hospital may be appropriate, their reluctance to convey was particularly evident through use of emotive terms such as ‘dragging’, ‘ripping’ and ‘kidnapping’ to describe removing someone from their home.

Participants generally described an approach aligned with delivering person-centred care (Kitwood, 1997), stressing the importance of dignity, respect and recognising the humanity of people with dementia. In describing how they attempted to build a rapport with people with dementia, paramedics recognised them as people with a wider social and historical context, rather than reducing them to their cognitive impairments (Mitchell, 2019). A minority of participants also described attempting to understand what was important to the person with dementia, and tried to ensure that they were given choices, even if limited, to promote that person’s agency. For example, one participant talked about the importance of enabling people with dementia to choose accessories such as handbags and hats to bring to hospital, reflecting the role that this can play in affirming identity for that person (Parry-Hughes & Dening, 2019).

Participants unanimously said that each call to a person with dementia was unique. However, Brooker and Latham (2016) caution that individualised care is not enough in itself to provide person-centred care, as individualised care plans can reflect the needs and priorities of the care-giver or organisation, not necessarily the person with dementia. This was evident in the way in which establishing rapport was described at times as a way to get someone to do what the paramedic wanted.

The infantilising of people with dementia, even for benign reasons, has been widely criticised as patronising and inappropriate (Mitchell, 2019; Mitchell & Agnelli, 2015). A minority of participants in this study compared how they work with people with dementia to working with children. However, these accounts, while clearly problematic, also expressed the participants’ desire to adapt communication to each individual to enable involvement.

Participants described a number of factors that affected the degree to which people with dementia were involved in decisions, including the level of cognitive impairment and/or distress, co-morbidities and the role of family or carers. Paramedics attempted to support, involve and negotiate with family members and carers. However, it was recognised that these relationships can also involve conflict, harm or tension (Peisah et al., 2006), and that care provision can be inadequate (Hughes, 2013).

Several participants mentioned difficulties that arise from the role of paramedics arriving as strangers and having to establish trust and make rapid assessments with limited information (Waldrop et al., 2015). There was also, however, recognition that the subjective approach of the paramedic is important, something that has been found to be the case among other professionals working with people with dementia (Brannelly, 2006). This subjective approach is informed by the values, skills, knowledge and human factors that the healthcare professional brings to the encounter (Collen, 2017).

The weighing up of multiple factors was frequently mentioned in relation to making best interest decisions *for* people assessed as lacking legal capacity. This process was generally in line with the Mental Capacity Act (2005). However, how competing factors were weighed varied between participants. This reflects the complexities and uncertainties often associated with paramedics’ decision making (Harenčárová, 2017). It also illuminates the fact that clinical decisions cannot be made in absence from values, as the weighting of clinical evidence, social and other factors involves value judgements (Seedhouse, 2005).

Participants’ values in this study were sometimes asserted by comparing their own approach to that of colleagues, reflecting a similar finding by Murphy-Jones and Timmons (2016) in relation to end-of-life care. An interesting split emerged, with some newly qualified paramedics complaining that longer-serving colleagues had lost empathy and longer-standing staff asserting that new staff lacked patience and life experience.

Participants discussed the way in which their role increasingly encompasses urgent rather than emergency care, with the need to make increasingly complex decisions that are not purely protocol led. This echoes ideas around changing role identity that Simpson et al. (2017) found were central to paramedic decision making in relation to older people who have fallen.

## Limitations

The sample size in this study was limited by pragmatic considerations, as it was designed as an initial exploratory study. It was not expected, therefore, that the study would achieve data saturation – the point at which increasing the sample no longer offers new insights (Bryman, 2016). However, the authors were also cautious about focusing on saturation as it is a concept usually aligned with grounded theory and it can therefore be problematic to try to graft it onto other methodologies (Caelli et al., 2003). Furthermore, as O’Reilly and Parker (2013) point out, it is worth considering whether saturation is ever truly reached as there are potentially new things that could be discovered in any study. The study was based in one UK ambulance service and so may not be transferable to other paramedic populations. This is particularly relevant given that it is unknown whether paramedics’ values are developed in relation to the culture of a particular ambulance trust. The nature of pre-hospital care is changing rapidly, so these findings are also likely to be specific to this moment (Murphy-Jones & Timmons, 2016).

The participants largely self-selected, and four out of seven disclosed that a family member had dementia. This added to the richness of discussion, and probably correlates to the high likelihood of families being affected by dementia (Prince et al., 2016). However, the data may have been affected by the predominance of those with personal experience of dementia.

The main theoretical limitation of the study is that it seeks to investigate the involvement of people with dementia, but relies on paramedics’ perspectives. This was due to the practical limitations of a small unfunded study. Future research on this topic would be strengthened by also investigating the perspectives of people with dementia and those close to them (Cashin et al., 2019).

## Conclusion

Decision making that involves people with dementia is often complex, raising ethical concerns as well as medical issues (Hughes & Baldwin, 2006). It can be especially challenging for paramedics who are attempting to establish good relationships and make sound decisions in a short amount of time.

This study found that paramedics are committed to providing high quality care that values people with dementia and attempts to understand things from their perspectives. However, the involvement of people with dementia in decisions about their care is sometimes limited by medical, social or clinician-dependent factors. In particular, this study highlights the impact of paramedics’ values and attitudes on their practice. Further research could examine how those values are formed and explore in more depth how they inform practice. The study also emphasises the impact of resources, including social care and community health teams, in providing safe, dignified care that respects the wishes and needs of people with dementia.

Finally, as paramedicine evolves to encompass more urgent and chronic care, paramedic education and training needs to include a focus on the communication skills and holistic decision making needed to ensure that person-centred care is at the centre of modern paramedic practice.

## Author contributions

Esme Choonara was the primary researcher and author, supervised by Julia Williams. Both authors contributed to the final manuscript. JW acts as the guarantor for this article.

## Conflict of interest

None declared.

## Ethics

Ethical approval for the study was granted by the University of Hertfordshire Health, Science and Technology Ethics Committee with Delegated Authority. Reference HSK/PGT/UH/03761.

## Funding

None.
